# Drug Utilization Evaluation of Erythropoietin at a Referral Teaching Hospital in Iran

**DOI:** 10.1155/2023/6685602

**Published:** 2023-11-07

**Authors:** Iman Karimzadeh, Hanieh Rasekh, Ava Karimian, Mojtaba Shabani-Borujeni, Afsaneh Vazin

**Affiliations:** ^1^Department of Clinical Pharmacy, School of Pharmacy, Shiraz University of Medical Sciences, Shiraz, Iran; ^2^Student Research Committee, Shiraz University of Medical Sciences, Shiraz, Iran

## Abstract

**Objectives:**

Drug utilization evaluation (DUE) studies aim to survey the appropriateness of drug use. DUE is an executive approach used to improve the use of medications as well as reduce the cost of treatment, ensure drug adequacy, and improve patient safety. The aim of this study was to evaluate the pattern of erythropoietin use, according to standard guidelines, in patients admitted to Namazi Hospital in Shiraz, Iran.

**Methods:**

In this descriptive, retrospective study, 230 patients were assessed. All patients who were hospitalized in different wards of Namazi Hospital, affiliated to Shiraz University of Medical Sciences, and received at least three doses of erythropoietin from September 2019 to March 2020 participated in this study. The following standard indicators of erythropoietin use were evaluated through reviewing medical charts of the cohort: drug dose, dosing intervals, route of administration, indication, monitoring of laboratory parameters, drug dose adjustment based on the response rate as well as target hemoglobin ≥12 g/dl, attention to major drug interactions, and administration of injectable or oral iron supplementation during treatment.

**Results:**

Most (65.2%) of the participants were male. The mean ± SD age of the patients was 47.55 ± 22.71 years. More than half (51.3%) of the included subjects were hospitalized in the nephrology ward. PDpoetin® and Cinnapoietin® were given to 52.6% and 47.4% of the study participants, respectively. Treatment of anemia due to chronic kidney disease was the most frequent indication of erythropoietin. The time interval of erythropoietin administration was three times a week for 68.3% of the patients. The most frequently administered weekly dose of erythropoietin was 12,000 units. The weekly dose, dose interval, and route of administration of erythropoietin were appropriate in 52.6%, 77.4%, and 100% of the patients, respectively. Dose adjustment based on the response rate, attention to major drug interactions as well as absolute-relative contraindications, and attention to the target hemoglobin ≥12 g/dl to decide whether or not to continue treatment were based on standard guideline in 98.1%, 98.7%, and 93% of the patients, respectively. The sum indexes of erythropoietin use were in line with standard guidelines in 75.84% of the cases.

**Conclusion:**

According to our results, in the setting of erythropoietin use in hospitals, physicians need more attention and education in areas such as selecting the proper dose of medication, correct indication of the drug, temporal arrangement of monitoring laboratory items, and the patient's need for iron supplements.

## 1. Introduction

Drug utilization evaluation (DUE) studies are intended to survey the appropriateness of drug usage. DUE is important to understand that improper use of drugs can have potential risks and additional costs for patients [1]. It is an executive approach used to improve the usage of medications as well as reduce the cost of treatment, ensure drug adequacy, and improve patient safety [2].

Erythropoietin (EPO) is a major factor in the growth of erythrocytes and acts as the main regulator of erythropoiesis by enhancing the survival, proliferation, and differentiation of erythroid stem cells and regulating the number of erythrocytes in the peripheral blood [3]. The main source of EPO production after fetal growth is the liver, while in adults, the main source of EPO production is the kidneys [4]. In severe anemia, the levels of blood serum EPO increase compared with the normal range. Tissue oxygen depletion is the most powerful stimulant for EPO production. Cells that control the synthesis of this hormone respond to changes in oxygen capacity, oxygen pressure, and the desire of blood for oxygen. In addition, local blood flow and cellular oxygen consumption may play a role in this process [5].

EPO is available in the pharmaceutical market as an injectable formulation. It is mainly indicated for the management of anemia caused by either EPO deficiency/insufficiency or resistance in different clinical settings such as chronic kidney disease (CKD), congestive heart failure, chemotherapy, and HIV [6]. Besides, EPO has also cardioprotective, renoprotective, and neuroprotective functions [7]. Due to the significant cost and different indications of EPO, determining its pattern of use in both outpatients and inpatients seems crucial. On the other hand, overuse and lack of appropriate monitoring as well as dose adjustment of this agent can increase the risk of several complications such as venous thromboembolism, stroke, hypertension, tumor progression, and even death. To our knowledge, there are no published data about this issue in Iran. Therefore, this study aimed to evaluate the pattern of EPO use and compare its indexes with standard instructions in a referral hospital in Iran.

## 2. Methods

The usage pattern of EPO was evaluated in this observational, retrospective study during 7 months from September 2019 to March 2020 in all wards of Namazi Teaching Hospital, affiliated to Shiraz University of Medical Sciences, Shiraz, Iran. This is a multispecialty, tertiary, and referral healthcare setting in the Southwest of Iran. It has more than 900 beds with above 30 specialty and subspecialty wards. All stages of the study were reviewed and approved by the Institutional Review Board and the Medical Ethics Committee of Shiraz University of Medical Sciences (Ethical ID: IR.SUMS.REC.1399.380).

The inclusion criteria were receiving at least three doses of EPO for any labeled/unlabeled indication, and hospital stay for at least one week. There was no limitation for the age and hospital ward of the participants, EPO type (alpha versus beta), and route of administration (subcutaneous versus intravenous). Patients who had received EPO for the management of methanol poisoning were not eligible for our study because this is currently an investigational indication of EPO, and there is no consensus about the standard dose, route of administration, and duration of treatment with EPO.

Under the supervision of a clinical pharmacist, a trained and qualified pharmacist collected the required information from each patient using a predesigned form by reviewing their relevant medical records as well as their hospital information system (HIS) records. The information was as follows (supplementary 1): Demographic and clinical characteristics including age, sex, weight, date of admission and discharge, past medical history, coadministered medications during hospitalization, and blood transfusion; paraclinical findings including cell blood count, hematocrit level, serum potassium level, serum creatinine level (only in CKD patients), serum iron level, transferrin saturation percentage, ferritin level, serum folate level, serum vitamin B12 level, blood pressure, and basal and average hemoglobin level before and during receiving treatment; and EPO dose, frequency as well as route of administration, brand name (PDpoetin® or Cinnapoietin®), duration of treatment, and possible dose adjustments if necessary.

In CKD patients, glomerular filtration rate (GFR) at the time of starting EPO was calculated based on the CKD-EPI equation [8]. Potential interaction between EPO and other coadministered medications were assessed by the Lexi-Interact online software. Adequate response to EPO was defined as an increase in hemoglobin level of 0.5 to 1.0 g/dL during a 1- to 2-week period of treatment. Being underresponsive and over-responsive to EPO were defined as less than 1 g/dL increase in hemoglobin level within 2 to 4 weeks and more than 1.0 g/dL during a 1- to 2-week period of treatment, respectively [9].

According to a checklist (supplementary 2), the studied indicators of EPO use were as follows: (1) drug dose, (2) intervals and frequency of administration, (3) route of administration, (4) monitoring clinical/paraclinical parameters at baseline and during treatment, (5) indication, (6) dose adjustment based on Hb response rate as well as target Hb (≥12 g/dl), (7) attention to the absolute and relative contraindications, (8) attention to the major drug interactions, (9) injectable or oral iron supplementation during treatment, and (10) required dose adjustment based on the response rate of Hb. Each inappropriate or appropriate index was scored as 0 or 1, respectively. The total score for EPO use indices was also reported by a trained and qualified pharmacist.

Standard EPO use indices in relation to the above items were extracted from reputable medical and pharmaceutical sources including Uptodate online; Applied Therapeutics: The Clinical Use of Drugs, 11th edition, 2018; Pharmacotherapy: A Pathophysiologic Approach 11th edition, 2020; Brenner and Rector's The Kidney, 11th edition, 2020; and KDIGO Clinical Practice Guideline for Anemia in Chronic Kidney Disease KDIGO, 2012.

All statistical analyses were performed using the SPSS software, version 20. The normality of the distribution of continuous data was determined by the Kolmogorov–Smirnov test. Normally- and not-normally distributed continuous data were reported as mean ± standard deviation (SD) and median, respectively. Categorical variables were reported as a percentage. Comparison of the appropriateness of each index of EPO use was performed using the Chi-square test. This test was also used to compare frequencies of achieving adequate response to EPO treatment and also the need for EPO dose adjustment between two brand names of EPO (PDpoetin® and Cinnapoietin®). *P* values less than 0.05 were considered as statistically significant.

## 3. Results

In this study, 230 patients with the mean ± SD age of 47.6 ± 22.7 years were recruited. About sixty percent (59.6%) of the participants were from 18 to 64 years old. More than half (65.2%) of the study population were male. The most common underlying diseases of the study population were CKD (56.1%), diabetes (11.3%), and heart failure (8.7%). Most patients were hospitalized in the nephrology (51.3%), emergency (16.1%), pediatric internal medicine (10.4%) wards, and intensive care unit (8.7%).

Laboratory parameters and different aspects of EPO use in the study population are shown in Table 1. In terms of EPO brand name, 52.6% and 47.4% of participants were treated with PDpoetin® and Cinnapoietin®, respectively. In more than three-fourths (77.4%) of the cohort, the interval of EPO administration was either three times or once a week. In all patients, EPO was administered subcutaneously. Management of anemia caused by CKD was the most frequent indication of EPO in the study population. The most common weekly dose of EPO used was 12,000 units. Serum ferritin, vitamin B12, and folate levels were measured in 91, 11, and 7 patients, respectively. In addition, 137 patients received oral folic acid. Iron (oral or parenteral) and vitamin B12 (oral) were given to 47 and 46 subjects, respectively. Apart from EPO, 97 patients received at least one unit of whole blood or packed cell.

Laboratory parameters including daily complete blood count (CBC), serum potassium level, blood pressure, and iron profile were evaluated during hospitalization. All the above laboratory parameters were monitored at least once for 54.3% of the patients. In more than three-fourths of the cohort (77.4%), the mean hemoglobin level during EPO treatment was less than 11 g/dl. Response to EPO treatment was adequate in more than half of the cohort (53.9%). Similarly, the dose of EPO was not changed in more than half of the study population (54.8%). The frequencies of achieving adequate response to EPO treatment (*P* = 0.165) and also the need for EPO dose adjustment (*P* = 0.152) were comparable between patients receiving PDpoetin® and Cinnapoietin®.

Regarding possible absolute or relative contraindications of EPO, all patients were evaluated for a history of anaphylaxis, uncontrolled hypertension, active thromboembolism, and active seizures. Only three patients with uncontrolled hypertension (mean systolic blood pressure greater than 160 mm Hg) had received EPO. In most patients (98.7%), no drug interaction with EPO was reported. Only three cases (1.3%) of type C interaction of EPO with thalidomide were reported in patients with multiple myeloma.

The mean ± SD of the length of hospital stay of the study population was 2.8 ± 1.8 weeks, with the minimum and maximum of 1 and 10 weeks, respectively. The survival rate for the study population was 77%.

A comparison of the total erythropoietin administration indices with the standard guidelines is shown in Table 2. The need for administering injectable or oral iron supplements during EPO treatment (70.5%), EPO dose (47.4%), and monitoring of laboratory and clinical parameters before and during EPO treatment (45.7%) were the most common usage indexes determined to be inappropriate as compared with the standard guidelines. Except for EPO dose (*P* = 0.23), indication (*P* = 0.11), and monitoring all required clinical and paraclinical parameters before and during the treatment (*P* = 0.11), the difference between the rate of other appropriate and inappropriate indexes of EPO use reached the level of statistical significance (*P* < 0.05). The mean ± SD sum of all indicators of EPO use in the study population was 75.8 ± 25.7. In other words, the rate of compliance of all ten indicators of EPO use with standard guidelines in this study was 75.8%.

## 4. Discussion

The present study aimed to evaluate the pattern of EPO usage in different wards of a referral and teaching hospital in Shiraz, Iran. EPO was selected for this DUE study because it is categorized as an essential medication based on the VEN analysis. Moreover, according to the ABC analysis, EPO fits into the B category in this hospital. Finally, as far as we know, different aspects of EPO use have not been studied so far in Iran.

In the present study, more than half (56.1%) of the participants received EPO to manage anemia related to CKD. It is not a surprising finding because CKD was the most common underlying disease of the study population. In addition, more than three-fourths (78.3%) of our patients had estimated GFR less than 60 ml/min/1.73 m^2^. Anemia becomes increasingly common as estimated GFR declines below 60 ml/min/1.73 m^2^ [10]. About 90 percent of CKD patients with estimated GFR less than 25 to 30 ml/min/1.73m2 have some degrees of anemia [11]. After CKD-associated anemia, the most common indications of EPO were anemia of chronic disease (38.3%) and chemotherapy-induced anemia (5.7%). The two primary FDA-approved indications for erythropoietin stimulating agents (ESA) use include anemia due to CKD and anemia caused by chemotherapy in cancer patients [12]. Accordingly, the FDA has approved the use of EPO (1993) and darbepoetin (2002) in patients with chemotherapy-induced anemia. It is necessary to note that darbepoetin was not available and utilized in our country at the time of performing this study.

In patients with CKD and chemotherapy-induced anemia, ESA is usually limited to patients with a hemoglobin level of less than 10 g/dL. This is mostly due to the risk of side effects, especially thromboembolic events, stroke, tumor progression, and even death if hemoglobin levels reach above 12 g/dL [12–14]. Accordingly, the KDIGO guidelines also recommend that the target hemoglobin level in the treatment of anemia due to CKD is 11.5 g/dL [9]. This is the major cause of inappropriate use of EPO in the present study, especially in nephrology wards. In more than one-third (45.2%) of the study population, the EPO dose was modified to achieve the target hemoglobin level. Over-response to EPO treatment was only identified in 6.5% of patients. Regular monitoring of hemoglobin level (at least once weekly until it becomes stable and then 1 to 2 times per month) and modifying the EPO dose accordingly, if required, can tremendously help the health care team to achieve the target hemoglobin level as well as minimize EPO overtreatment, extra costs, and its dose-dependent side effects.

Iron deficiency is the main contributing factor in ESA resistance or failure. Therefore, monitoring and correction (if necessary) of iron deficiency by either oral or parenteral iron before starting EPO is recommended. In this regard, for example, Nobahar et al. in a case-control study found that administering oral/parenteral iron first, followed by EPO, could manage anemia in hemodialysis patients more effectively than EPO or iron supplementation alone [15]. However, these measures were not considered before prescribing EPO in about 70% of our cohort. This may be partially due to the high turnover of patients in internal wards and the preference of physicians to defer measuring iron profile and other relevant laboratory tests to the outpatient setting. Therefore, physicians, especially nephrologists, should be more vigilant about the appropriate time of initiating EPO, checking iron profile before initiating EPO, and close monitoring of its response.

For various indications of EPO, its usual dose is 50–100 units/kg three times a week. In the case of chemotherapy-induced anemia, EPO usually starts at 40,000 units per week and can be increased up to 60,000 units per week [16]. The dose of EPO given into our patients ranged from 4,000 units to 16,000 units per week with the mean dose of 10,704 units per week. No patient received EPO more than 60,000 units per week, which is generally considered as its maximum allowable weekly dose. According to the results of a cross-sectional study in 4 community-based, university-affiliated nursing homes in the United States, the mean ± SD weekly dose of epoetin alpha was 22,625 ± 21,232 units [17]. Obviously, appropriate EPO dose should be selected and adjusted based on its indication and hemoglobin target level.

In our study, most patients (68.3%) were given EPO three times a week. The frequency of administration of EPO was twice a week in 22.6% of our patients. This frequency of EPO administration was not generally recommended in the literature and can be considered to be inappropriate. In a multicenter, retrospective, observational study on 237 critically ill patients admitted to ICU in the United States who were receiving EPO alpha, the most common dosing frequency was 3 times weekly (35.9%) [18]. Some ESA formulations including darbepoetin and methoxy polyethylene glycol-EPO beta have been prepared which provided the possibility of weekly and even monthly administrations, respectively. Nevertheless, these agents were not routinely available in our country at the time of conducting this study.

As to the route of administration, EPO was injected subcutaneously in all our patients by the nurse in charge. Since the present study methodology was retrospective, it was not feasible to determine whether the technique of subcutaneous injection by the nursing staff was in accordance with the standard guideline and correct. In addition to the subcutaneous route, EPO can also be given intravenously. However, due to the ease of intravenous administration in individuals under hemodialysis, lack of pain or stinging sensation, and also the risk of rare but serious complication of pure red cell aplasia that occurs more frequently in subcutaneous administration of EPO, intravenous injection appears to be a better option than subcutaneous injection in hemodialysis patients [13]. However, due to the difference in half-life, intravenous EPO doses were on average 25% higher than the subcutaneous ones for achieving equivalent hemoglobin responses [19]. Therefore, modifying the EPO dose should be taken into account in the case of changing the administration route from subcutaneous to intravenous.

As to the brand name, more than half (52.6%) of EPO formulation administered in this study was PDpoetin®. This is an EPO alpha product manufactured by the Pooyesh Darou Pharmaceuticals in Iran. According to Iranian Ministry of Health Pharma Statistics during the first eight months of 2018, 58.12% and 48.85% of EPO 2,000 and 4,000 units dosage forms were manufactured by this company, respectively [20]. The remaining patients in our study (47.4%) were given Cinnapoietin®, which is an EPO beta product of CinnaGen pharmaceutical company in Iran. Apart from the proportion of each brand name of EPO in Iranian pharmaceutical market, both PDpoetin® and Cinnapoietin® have documented evidence, as published articles, demonstrating their clinical effectiveness and safety in hemodialysis patients [21, 22]. Compared to EPO alpha, EPO beta has a higher molecular weight, but lower number of sialylated glycan residues. Therefore, EPO beta may benefit from a longer terminal elimination half-life [23]. However, the clinical relevance of these structural and pharmacokinetics differences is not completely understood. In this regard, at least five clinical studies have compared the efficacy of EPO alpha and beta. For example, a longitudinal, retrospective study on hemodialysis patients in the UK demonstrated that despite both EPO alpha and beta reach target hematocrit levels, it is achieved with significantly lower doses of EPO beta [24]. Similarly, results of a randomized, active-controlled, double-blind, parallel, and noninferiority trial in Iran about the comparison of Cinnapoietin® with Eprex® suggested that Cinnapoietin® was noninferior to Eprex® in the treatment of anemia in hemodialysis patients [22]. On the other hand, findings of two other studies in Pakistan and Japan favored EPO beta over alpha in the management of anemia in patients with CKD under or not under hemodialysis [25, 26]. In contrast, a prospective, observational study in patients with CKD under hemodialysis in Indonesia reported that EPO alpha is more effective in achieving the goal hemoglobin level compared to EPO beta [27]. Although it was not the main goal of the present study and our research methodology was not also much appropriate for this comparison, the rate of achieving adequate response to EPO and the need for EPO dose adjustment based on hemoglobin levels did not differ significantly in recipients of PDpoetin® and Cinnapoietin®. Notably, there is currently no head-to-head clinical trial to compare the different aspects including safety and efficacy of Cinnapoietin® and PDpoetin®.

EPO treatment can be associated with several side effects such as flu-like syndrome, hypertension, thrombosis, seizure, stroke, and hypokalemia, the most common of which is hypertension. The incidence rate of EPO-associated hypertension is estimated to be 10–15%. The increase in blood pressure by EPO can be especially harmful to patients with underlying cardiovascular or kidney diseases [28]. Therefore, blood pressure should be measured at baseline and also monitored closely during the EPO treatment. K/DOQI guideline for the management of anemia in the setting of CKD does not recommend withholding therapy in the case of elevated blood pressure; instead, it advocates the use of antihypertensive agents and dialysis to control blood pressure [29]. In contrast, FDA-approved product labeling recommends that EPO should not be used in those with uncontrolled blood pressure. Only three patients (1.3%) in our study had mean systolic blood pressure above 160 mmHg during EPO treatment. It is noteworthy that considering this cut-off value for blood pressure as a contraindication of EPO use is mostly arbitrary and center-dependent. In terms of possible relation of hypokalemia with EPO use, at least one study on patients undergoing continuous ambulatory peritoneal dialysis in South Korea implicated that neither EPO administration nor its dose had a significant association with hypokalemia defined by an average serum potassium level of less than 3.5 mEq/L [30]. Finally, there were no other cases of absolute or partial contraindication of EPO (e.g., pure red cell aplasia and documented hypersensitivity reactions to EPO [31]) in the study population.

In three patients with multiple myeloma who received thalidomide, type C interaction with EPO was observed in our study. This category of interaction should be monitored if these drugs are taken simultaneously. The mechanism of this interaction increased the risk of venous thromboembolism such as deep vein thrombosis or pulmonary embolism. However, prophylaxis of thromboembolism with anticoagulants in these conditions is unnecessary and not recommended [32]. The same interaction has been reported with other agents of this class including lenalidomide and pomalidomide. Note that the above identified interaction in our study is just potential, not clinically relevant.

The main limitations of this study are as follows: (1) The study was performed only in one hospital. Therefore, center bias may exist, and our results cannot be generalizable; (2) the retrospective method of this study prevented access to all required information. The possible defects in the medical chart of patients may highlight this drawback; and (3) it is not feasible to give feedback and correct the identified deviations of EPO use from standard guideline by pharmacists because this study was retrospective and observational. It has been reported that implementation of a drug-utilization management program by clinical pharmacists was associated with a significant decrease in inappropriate EPO prescription along with significant cost-savings [33]. Other studies from Iran also confirmed the beneficial roles of clinical pharmacists in improving the pattern of the use of high-cost medications at hospitals [34].

## 5. Conclusion

Our results showed that EPO usage was not fully in line with standard guidelines due to administration of incorrect dose, administration of the medication outside its labeled/off-labelled indications, and lack of required paraclinical monitoring including daily CBC, potassium serum level, blood pressure, and iron profile. It seems that heavy workload, inadequate academic as well as continuing medical education programs for physicians and nurses, and lack of regular surveillance systems can be the main reasons for inappropriate use of EPO in our hospital. Apart from training physicians and nurses on proper prescription and administration, regular and active presence of clinical pharmacists in different wards as well as drug and therapeutic committee of the hospital can improve the pattern of EPO usage. Therefore, multidisciplinary strategies should be investigated in future clinical studies to achieve maximum improvement in different aspects of EPO use in hospitals.

## Figures and Tables

**Table 1:** Laboratory factors and erythropoietin characteristics of the study population (*n* = 230).

Variable	Value
Mean basal hemoglobin (g/dl)	8.8 ± 1.9
*Mean hemoglobin during EPO treatment (g/dl) (%)*	
(i) Less than 11	178 (77.4)
(ii) Between 11 and 12	29 (12.6)
(iii) More than 12	23 (10)
Mean hematocrit during EPO treatment (%)	28.9 ± 5.2
Mean serum potassium during EPO treatment (mEq/L)	4.3 ± 0.5
Mean systolic blood pressure (mm Hg)	119.5 ± 15.4
Mean diastolic blood pressure (mm Hg)	73.50 ± 7.6
*Glomerular filtration rate (ml/min/1.73 m* ^*2*^)	
(i) Less than 60	101 (78.3)
(ii) Equal or more than 60	28 (21.7)
Transferrin saturation, *n* (%)	78 (18.5)
Serum ferritin level (ng/ml)	5.517
*Prescribing iron supplements, n (%)*	
(i) Injectable form	34 (14.8)
(ii) Oral form	13 (5.6)
Prescribing folic acid tablets, *n* (%)	137 (59.6)
Prescribing vitamin B12 ampoules, *n* (%)	46 (20)
EPO brand, *n* (%)	
(i) PDpoietin® (EPO alpha)	121 (52.6)
(ii) Cinnapoietin® (EPO beta)	109 (47.4)
Indication for prescription, *n* (%)	
(i) Anemia due to chronic kidney disease	129 (56.1)
(ii) Anemia due to chemotherapy	13 (5.7)
(iii) Anemia due to chronic disease	88 (38.3)
*Number of EPO injections per week, n (%)*	
(i) Once a week	21 (9.1)
(ii) Twice a week	52 (22.6)
(iii) Three times a week	157 (68.3)
*Response to EPO, n (%)*	
(i) Adequate response	124 (53.9)
(ii) Over response	15 (6.5)
(iii) Under response	91 (39.6)
*Dose modification of EPO, n (%)*	
(i) No dose change	126 (54.8)
(ii) Dose reduction	15 (6.5)
(iii) Dose increase	89 (38.7)

EPO: erythropoietin.

**Table 2:** Comparison of total erythropoietin administration indices with standard instructions in the study population (*n* = 230).

Index	Appropriate (%)	Inappropriate (%)	*P* value
Dose	52.6	47.4	0.2293
Intervals and frequency of administration	77.4	22.6	<0.0001
Route of administration	100	0	<0.0001
Indication	56.1	43.9	0.1056
Monitoring of laboratory and clinical parameters before and during treatment	54.3	45.7	0.1085
Proportion of dose adjustment based on response rate	98.1	1.9	<0.0001
Attention to the target Hb ≥ 12 to decide whether or not to continue treatment	93	7	<0.0001
Attention to absolute and relative contraindications	98.7	1.3	<0.0001
Monitoring drug interactions	98.7	1.3	<0.0001
Administration of injectable or oral iron supplements during treatment (only in CKD patients)	29.5	70.5	<0.0001

Hb: hemoglobin, CKD: chronic kidney disease.

## Data Availability

The datasets generated and/or analyzed during the present study are not publicly available since they contain information that could compromise the privacy of research participants but are available from the corresponding author on reasonable request.

## Supplementary Materials

Supplementary Materials Supplementary 1 shows data collection form for the study population. It comprises demographic information of patients (name, gender, age, weight, date of admission, and hospitalized ward), laboratory findings (serum iron, hematocrit, transferrin, ferritin level, serum folate, serum B12, serum creatinine level, and mean hemoglobin value), and clinical characteristics (such as ESA brand name, duration of treatment, dosing frequency, dose modification, route of administration, past medical history, drug history and iron, B12, and folic acid supplementation). Supplementary 2 lists indicators related to EPO use in the study population. These indicators include drug dose, intervals and frequency of administration, route of administration, monitoring clinical/paraclinical parameters at baseline and during treatment, indication, dose adjustment based on Hb response rate as well as target Hb (≥12 g/dl), attention to the absolute and relative contraindications, attention to major drug interactions, injectable or oral iron supplementation during treatment, and required dose adjustment based on the response rate of Hb.
