# Supplementary material for: Drug Utilization Evaluation of Erythropoietin at a Referral Teaching Hospital in Iran
Source: Adv Pharmacol Pharm Sci. 2023 Nov 7;2023:6685602. doi: 10.1155/2023/6685602 (PMC10645503; doi:10.1155/2023/6685602)
Supplement: Supplementary Materials — Supplementary 1 shows data collection form for the study population. It comprises demographic information of patients (name, gender, age, weight, date of admission, and hospitalized ward), laboratory findings (serum iron, hematocrit, transferrin, ferritin level, serum folate, serum B12, serum creatinine level, and mean hemoglobin value), and clinical characteristics (such as ESA brand name, duration of treatment, dosing frequency, dose modification, route of administration, past medical history, drug history and iron, B12, and folic acid supplementation). Supplementary 2 lists indicators related to EPO use in the study population. These indicators include drug dose, intervals and frequency of administration, route of administration, monitoring clinical/paraclinical parameters at baseline and during treatment, indication, dose adjustment based on Hb response rate as well as target Hb (≥12 g/dl), attention to the absolute and relative contraindications, attention to major drug interactions, injectable or oral iron supplementation during treatment, and required dose adjustment based on the response rate of Hb. [file 6685602.f1.zip › Supplementary 2.docx]

| • Dose:  **Supplementary 2. Indicators related to erythropoietin use in the study population**   - Starting dose: 50-100 unit/kg per dose € - Maximum dose: 60,000 units per week € - Others: |
| --- |
| • Frequency of administration:   - Once a week € - Three times a week € - Others: |
| • Route of administration:   - Subcutaneous € - Intravenous € |
| • Prescription indication:   - Anemia due to chronic kidney disease in patient with hemoglobin level less than 10 g/dl € - Anemia of chronic disease in patient with hemoglobin level less than 10 g/dl € - Anemia caused by zidovudine in patients with HIV € - Anemia associated with chemotherapy in patient with hemoglobin level less than 10 g/dl € - Symptomatic anemia in the context of myelodysplastic syndrome € - Anemia due to preterm delivery in infants (prematurity) € - Minimizing allogeneic blood transfusion after surgical procedures in patients with baseline hemoglobin less than 10 g/dl € |
| • Monitoring of laboratory parameters before starting and during treatment:   - Blood cell count € - Blood pressure € - Iron profile € - Serum potassium € |
| • Adjusting dose based on the response rate:   - Normal response € - Over response: Increase of more than one Hb unit during two weeks € - Under response: Increase of less than one Hb unit in a period of four weeks € - In the conditions of over response and under response, the dose of erythropoietin should be reduced by about 25% and increased by about 50%, respectively € |
| • Considering target Hb ≥ 12 g/dl to decide whether continuing or stopping the treatment:  Yes € No € |
| • Drug interactions:   - Carfilzomib € - Lenalidomide € - Thalidomide € - Pomalidomide € |
| • Absolute and relative contraindications:   - Sensitivity to human albumin or cells derived from mammals € - Uncontrolled blood pressure € - Active thromboembolism € - History of seizures or active seizures € - Documented history of pure red aplasia € - Porphyria € |
| • Administration of iron supplements (IM, IV, Oral) during treatment only in CKD patients:  Yes € No € |
