# Supplementary material for: Drug Utilization Evaluation of Erythropoietin at a Referral Teaching Hospital in Iran
Source: Adv Pharmacol Pharm Sci. 2023 Nov 7;2023:6685602. doi: 10.1155/2023/6685602 (PMC10645503; doi:10.1155/2023/6685602)
Supplement: Supplementary Materials — Supplementary 1 shows data collection form for the study population. It comprises demographic information of patients (name, gender, age, weight, date of admission, and hospitalized ward), laboratory findings (serum iron, hematocrit, transferrin, ferritin level, serum folate, serum B12, serum creatinine level, and mean hemoglobin value), and clinical characteristics (such as ESA brand name, duration of treatment, dosing frequency, dose modification, route of administration, past medical history, drug history and iron, B12, and folic acid supplementation). Supplementary 2 lists indicators related to EPO use in the study population. These indicators include drug dose, intervals and frequency of administration, route of administration, monitoring clinical/paraclinical parameters at baseline and during treatment, indication, dose adjustment based on Hb response rate as well as target Hb (≥12 g/dl), attention to the absolute and relative contraindications, attention to major drug interactions, injectable or oral iron supplementation during treatment, and required dose adjustment based on the response rate of Hb. [file 6685602.f1.zip › Supplementary 1.docx]

**Supplementary 1. Data collection form of the study population**

| ***Paraclinical Findings*** | ***Patient demographics*** |
| --- | --- |
| HCT(*%*): | Name: |
| Serum iron($\mu g/dl)$: | Gender (Male/Female): |
| TIBC ($\mu g/dl)$: | Age (y):   - <18 € - 18 – 64 € - 65 and older € |
| Transferrin saturation(*%*): |  |
| Ferritin level($g/dl)$: |  |
| Serum folate($ng/ml)$: | Weight (kg): |
| Serum vit B_12_($pg/ml)$: | Date of admission: |
| Blood pressure(*mmHg*): | Medical record number: |
| Serum Cr level in CKD patient ($mg/dl):$ | Hospitalized ward: |
| Mean Hb value($g/dl)$:   - < 11 € - 11-12 € - $\geq$12 € |  |

| ***Clinical characteristics*** |
| --- |
| ESA brand name:   - $\mathrm{Cinnapoietin}^{®}$ € - $\mathrm{PDpoetin}^{®}$ € |
| Duration of ESA treatment: |
| Dosing frequency of ESA:   - Once a week € - Twice a week € - Three times a week € |
| Weekly dose of ESA (units/week): |
| ESA dose modifications:   - Dose increase € - Dose reduction € - No dose adjustment € |
| Route of ESA administration:   - Intravenous € - Subcutaneous € |
| Past Medical history:   - Primary hypertension € - Diabetes mellitus € - Acute kidney injury (AKI) € - Chronic kidney disease (CKD) € - Under dialysis (hemodialysis or peritoneal dialysis) • Yes € • No € - Myocardial infarction € - Ischemic stroke € - Congestive heart failure(CHF) € - COPD, Asthma**,** Pneumonia € - Gastrointestinal bleeding € - Cancer € - Other diseases: |
| Drug history: |
| Co-administered medications: |
| Iron supplementation (IV, Oral or None): Yes € No € |
| Vit B12 supplementation (IM, Oral or None): Yes € No € |
| Folic acid supplementation: Yes € No € |
| RBC Transfusion: Yes € No € |
